# A Thorough Investigation of the Microbiological, Physicochemical, and Sensory Properties of Ewe’s Yoghurt Fermented by a Selected Multi-Strain Starter Culture

**DOI:** 10.3390/foods12183454

**Published:** 2023-09-15

**Authors:** Giuliana Garofalo, Marialetizia Ponte, Gabriele Busetta, Marco Tolone, Adriana Bonanno, Baldassare Portolano, Raimondo Gaglio, Hüseyin Erten, Maria Teresa Sardina, Luca Settanni

**Affiliations:** 1 Department of Agricultural, Food and Forest Sciences, University of Palermo, Viale delle Scienze, Bldg. 5, 90128 Palermo, Italy; giuliana.garofalo01@unipa.it (G.G.); marialetizia.ponte@unipa.it (M.P.); gabriele.busetta@unipa.it (G.B.); marco.tolone@unipa.it (M.T.); adriana.bonanno@unipa.it (A.B.); baldassare.portolano@unipa.it (B.P.); mariateresa.sardina@unipa.it (M.T.S.); luca.settanni@unipa.it (L.S.); 2 Department of Food Engineering, Faculty of Engineering, Çukurova University, Adana 1330, Turkey; herten@cu.edu.tr

**Keywords:** ewe’s yoghurt, milk starter culture, lactic acid bacteria, antioxidant capacity, sensory traits

## Abstract

This work was carried out with the aim to investigate the microbiological, physicochemical, and sensory properties of an innovative yoghurt produced from ewe’s milk. Experimental yoghurt productions were performed with a commercial freeze-dried starter preparation and a natural milk starter culture (NMSC) of *Streptococcus thermophilus* and *Lactobacillus delbrueckii*. The two yoghurts did not differ for colour parameters, showing an average value of lightness, redness, and yellowness of 94.99, −3.74, and 9.37, respectively. The yoghurt produced using the NMSC as a fermenting agent was characterised by a significantly lower fat percentage and a higher antioxidant potential than commercial starters. Microbiological analysis confirmed the safety of the final product and a level of living lactic acid bacteria of 10^8^ CFU/g. Sensory analysis revealed some differences among yoghurts regarding unpleasant odour, homogeneity, and persistence in the mouth, but the yoghurt processed with NMSC was more appreciated. Thus, the production of ewe’s yoghurt fermented by a selected multi-strain starter culture represents an interesting strategy to enlarge the functional ovine dairy product portfolio.

## 1. Introduction

Yoghurt is the most popular fermented milk product [1], and its unique properties are attributed to the symbiotic fermentation of *Streptococcus thermophilus* and *Lactobacillus delbrueckii* subsp. *bulgaricus* [2,3]. The nutritional value of this fermented milk product determines its worldwide consumption [4]. Indeed, yoghurt consumption can help people meet their nutritional needs for various nutrients, such as calcium, potassium, phosphorus, and vitamins B2 and B12 [5]. For this reason, yoghurt is generally known to contribute to the overall quality of the diet [6] and provide health benefits [4]. In some Asian countries, yoghurt is even used in folk medicine [7]. This product can also lower the risk of cardiovascular diseases and support bone health, especially for the elderly [8,9]. Moreover, yoghurt-fermenting bacteria express functional lactase, helping the digestibility of yoghurt compared to milk in intolerant subjects [10]. More than twenty years ago, Kailasapathy et al. [11] provided the first evidence of the positive impact of yoghurt consumption on the gut microbiota, which is associated with a reduced risk of gastrointestinal disease. Thus, the FAO/WHO recommended daily yoghurt intake due to its rich nutritional profile and its therapeutic and general benefits on the human body [12].

Yoghurt is usually made from cow’s milk. However, in several European and non-European countries, the milk from other species, such as sheep or goats, is processed into yoghurt [2,13]. In countries where climatic and soil conditions are not favourable for cow farming, the use of different types of milk would be a suitable alternative to cow’s milk to produce yoghurt. These conditions characterise Sicily, a southern Italian island region where sheep breeding is more common than cattle breeding (https://www.istat.it/, accessed on 27 July 2023). Ewe’s milk has several beneficial attributes over cow’s milk, providing higher levels of proteins, lipids, minerals, and vitamins essential for human health [14]. Several studies suggested that high levels of protein and fat positively affect the production of fermented milk products [15,16,17], contributing to increased firmness, cohesiveness, and viscosity of the yoghurt [18]. In particular, the viscosity of yoghurt may be useful in preserving probiotic cultures throughout the gastrointestinal tract. They offer added protection to probiotics in the stomach [19], as well as during the commercial storage period [20]. Furthermore, ewe’s milk yoghurt exhibits desirable flavour properties, such as the creamy-sour attribute appreciated by many consumers [21].

This work was carried out with the aim to develop an ewe’s milk yoghurt adapted to cow’s milk yoghurt technology. The goal was to expand the dairy Sicilian portfolio by targeting modern consumers who are increasingly interested in functional foods with higher nutritional values and health benefits. A natural milk starter culture (NMSC) was created using selected strains of *S. thermophilus* and *L. delbrueckii* isolated from typical Sicilian ewe’s dairy products. The use of NMSC is considered important to better link the novel product with the Sicilian area and to avoid the taste homologation phenomena determined by commercial starter cultures [22]. The resulting yoghurts were analysed for hygienic safety, physicochemical traits, and antioxidant capacity. Moreover, the yoghurts were also assessed for their sensory properties to determine how appealing the product is to consumers.

## 2. Materials and Methods

### 2.1. Raw Materials and Starter Cultures

Yoghurt was prepared using bulk milk of the “Valle del Belíce” breed, an indigenous Sicilian sheep species. Raw milk was obtained from several dairy farms located in Agrigento province (Cammarata, Italy) and was then transformed at “Cooperativa Agricola Tumarrano” (Cammarata, Italy). The experimental plan of this study consisted of two different ewe’s milk yoghurt productions by applying the technology typically followed in the production of cow’s yoghurt. The first production (EY-1) was performed using the commercial freeze-dried starter culture YODX091 (ALCE s.r.l., Quistello, MN, Italy), composed of *S. thermophilus* and *L. delbrueckii* spp. *bulgaricus* (one strain each), while a second production (EY-2) was carried out with the addition of a multi-strain NMSC developed in this study.

### 2.2. Preparation of Natural Milk Starter Culture

The NMSC used in this study included the following strains: *S. thermophilus* (PON244) and *L. delbrueckii* (WT601). These strains, selected for their high dairy performances such as acidification capacity, autolysis kinetics, diacetyl production, and antibacterial activity belonged to the collection of the laboratory of agricultural microbiology at the University of Palermo and were previously isolated from traditional PDO Sicilian cheeses [23,24]. Briefly, both strains were refreshed in their optimal growth media. *S. thermophilus* PON244 was reactivated in M17 medium [25], while *L. delbrueckii* WT601 was reactivated in MRS medium [26]. Both strains were incubated at 40 °C for 48 h. After growth, strains were subjected to two consecutive washes in Ringer’s solution (Sigma-Aldrich, Milan, Italy). Each washing step was followed by centrifugation at 6000× *g* for 2 min using a Heraeus Biofuge Pico (Kendro Lab, Waltham, MA, USA) in order to remove any residue of growth medium. The washed cells were inoculated at 10^6^ colony-forming units (CFU)/mL into ovine whole-fat UHT milk (Leeb Vital, Wartberg an der Krems, Austria) and incubated at 40 °C for 24 h. NMSC containing both *S. thermophilus* and *L. delbrueckii* at approximately 10^9^ CFU/mL, as verified by plate count, was used for yoghurt production.

### 2.3. Yoghurt Production and Sample Collection

**Figure 1:**
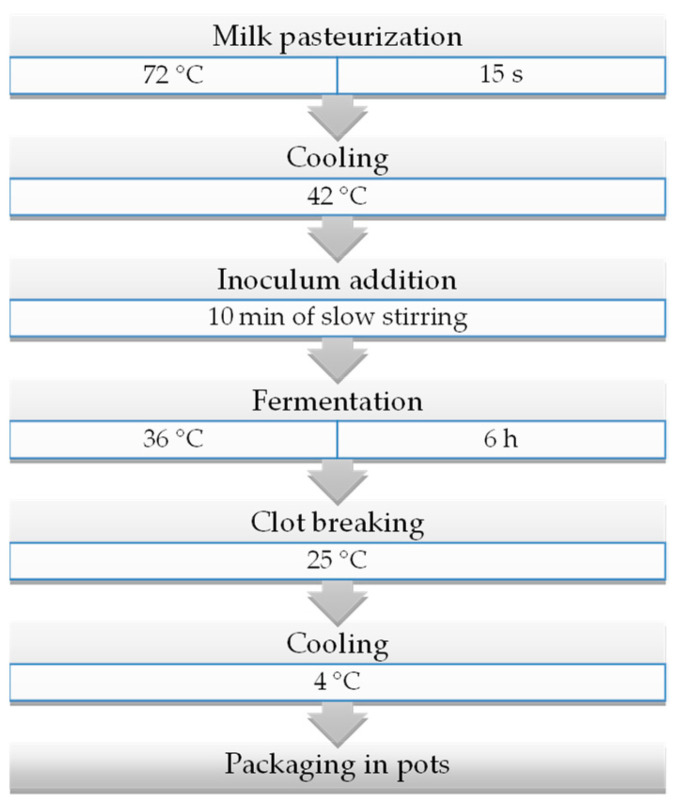
Flow chart of ewe’s yoghurt.

Yoghurts were prepared according to the production protocol indicated by Gaglio et al. [27]. Briefly, a volume of 100 L of raw milk was pasteurised at 72 °C for 15 s in a stainless-steel Comat PS 20351 pasteuriser (Bellizzi, SA, Italy) and cooled at 42 °C. The entire bulk milk was then transferred into an automatic Due Ci Inox s.r.l. yoghurt maker (Guastalla, RE, Italy). After cooling, starter cultures were directly inoculated, and the milk was stirred for 10 min. Fermentation took place at 38 °C for 6 h and was stopped at pH 4.5 [28]. Finally, the yoghurt was packaged into 120 mL plastic pots with an air-tight cap (FD Store s.r.l., Vignola, Italy) and stored at 4 °C for 3 d before distribution. Figure 1 provides an overview of the production protocol followed.

Both trials (EY-1 and EY-2) were repeated after two weeks (two independent replicates). Raw milk (RM), pasteurised milk (PM), inoculated milk (IM), and yoghurt (Y) after 3 d of refrigerated (4 °C) storage were collected for analyses.

### 2.4. Microbiological Analysis

All samples collected before and after yoghurt production were microbiologically evaluated to enumerate total mesophilic microorganisms (TMM) by spreading on plate count agar (PCA), incubated aerobically at 30 °C for 72 h, and thermophilic rod and coccus-shaped lactic acid bacteria (LAB) after pouring on de Man-Rogosa-Sharpe (MRS) agar acidified to pH 5.4 with lactic acid (5 mol/L) and M17 agar, respectively, incubated anaerobically in anaerobic jars (AnaeroGen, Thermo Fisher Scientific, Waltham, MA, USA) at 42 °C for 48 h. Fungal growth was inhibited by adding chloramphenicol (1 mg/L) to both media. Raw and pasteurised milk samples were also analysed for the presence of the main four food-borne pathogens in compliance with Commission Regulation (EC) No 2073/2005 [29] on microbiological criteria for foodstuffs: *Escherichia coli* and coagulase-positive staphylococci (CPS) were analysed as process hygiene criteria applying ISO 16649-1 [30] and ISO 6888-2 [31], respectively; *Salmonella* spp. and *Listeria monocytogenes* were analysed as food safety criteria by spreading on Hektoen enteric agar (HEA) and on *Listeria* selective agar base (LSAB) added with SR0140E supplement, respectively, both incubated at 37 °C for 24 h. All media and supplements were purchased from Oxoid.

The plate count method was applied after serial decimal dilutions. Aliquots of 1 mL of liquid samples (RM, PM, and IM) were diluted with 9 mL of sterile 0.9% (*v*/*v*) Ringer’s solution, while 15 g of yoghurt just after production and after 24 h of refrigerated storage were first homogenised in 135 mL of sodium citrate (2% *v*/*v*) solution in a stomacher (Bag Mixer 400; Interscience, Saint Nom, France) for 2 min at the highest speed and then serially diluted as described for liquid samples. Bacterial enumerations were carried out in duplicate (technical repeats) for each trial. The results were expressed as CFU per mL (liquid samples) or gram (solid samples).

### 2.5. Physicochemical Analysis

Milk samples before processing were analysed for pH, lactose, fat, protein, and urea by the infrared method (Combi-Foss 6000, Foss Electric, Hiller’d, Denmark). The colour of milk and yoghurts was evaluated in duplicate using a Minolta Chroma Meter CR-300 (Minolta, Osaka, Japan), which measures values of lightness (L* = 0/100, from black to white), redness (a* = −a/+a, from green to red) and yellowness (b* = −b/+b, blue to yellow), according to the CIE L*a*b* system [32]. The values of L*, a*, and b* were used for the calculation of chroma [(a*^2^ + b*^2^)^0.5^], measuring the colour intensity or saturation, hue angle [tan−1(b*/a*)], as a measure of colour tone, and the whiteness index [100−((100−L*)^2^ + a*^2^ + b*^2^)^0.5^], according to Vargas et al. [33]. Total colour change (ΔE) after yoghurt production was calculated as ΔE = [(ΔL*)^2^ + (Δa*)^2^ + (Δb*)^2^]^0.5^, where ΔL*, Δa*, and Δb* are the differences of L*, a*, and b*, respectively, between milk and yoghurt.

All yoghurt samples were freeze-dried and analysed for centesimal chemical composition [dry matter (DM), protein (N × 6.38), fat, and ash content] in accordance with FIL-IDF standards [34,35,36].

### 2.6. Oxidation Products and Antioxidant Capacity

The extracts of lyophilised samples were prepared following the protocol described by Rashidinejad et al. [37] with minor modifications. Briefly, each lyophilised yoghurt sample (0.5 g) was dissolved in a 95% aqueous methanol solution (25 mL) with 1% (*v*/*v*) HCl. The suspension was mixed by vortex for about 30 s and then held at 40 °C in an ultrasonic water bath (LBS1 Sonicator; Falc Instruments, Treviglio, Italy) for 30 min, during which it was mixed by vortex for 5–10 s every 10 min. The resulting suspension was cooled, filtered with linen cloth, centrifuged at 7000× *g* at 9 °C for 10 min, and finally kept at −18 °C until analysis. Extracted samples were analysed in duplicate for antioxidant properties, measuring the antioxidant capacity and the indexes of primary and secondary lipid oxidation.

Total antioxidant capacity in extracted yoghurt samples was assessed by the Trolox equivalent antioxidant capacity (TEAC) assay, according to a published protocol [37], as modified by Bonanno et al. [38]. TEAC is a discoloration assay aimed at evaluating the radical scavenging ability of the samples with the use of the [2,2′-azinobis(3-ethylbenzothiazoline-6-sulphonic acid)] (ABTS) radical cation (ABTS•+) and Trolox as standards [39]. To obtain the ABTS radical cation, equal volumes of a 14 mM ABTS aqueous solution and 4.9 mM K_2_S_2_O_8_ were mixed and placed in the dark for 16 h at room temperature. ABTS radical cation solution was diluted in 5 mM PBS solution (phosphate buffered saline, pH 7.40) until it reached an absorbance of 0.795 (±0.020) at 734 nm using a Hach DR/4000 U spectrophotometer (Hach Company, Ames, Iowa, USA). The absorbance reading of a mixture of 150 µL of PBS with 2850 µL of a diluted ABTS radical cation solution was recorded at 734 nm immediately (as white sample at 0 min) and after incubation at 30 °C for 6 min (as white sample at 6 min). Similarly, the absorbance of the 150 µL solution of each extracted sample mixed with 2850 µL of the dilute ABTS radical cation solution was read at 734 nm after 6 min of incubation at 30 °C. The absorbance values were used to determine the percentage decrease of the absorbance due to decolorization, calculated by comparison with the absorbance obtained with PBS. Solutions of Trolox in PBS (0–2.5 mM) were used to construct the calibration curve (R^2^ = 0.99), and the results are expressed in mmol Trolox/kg DM.

The oxidation of yoghurt fat was estimated by determining in duplicate the peroxide value (POV, mEq O_2_/kg fat), expressing the primary lipid oxidation [40], and the thiobarbituric acid reactive substances (TBARS, μg malonylaldehyde (MDA)/kg DM) as secondary lipid oxidation products, in line with the method described by Tarladgis et al. [41] and modified by Mele et al. [42]. Briefly, TBARS analysis was conducted on 2 g of lyophilised yoghurt, which was mixed with an 8 mL aqueous solution of phosphate buffer at pH 7 by vortex. After that, 2 mL of a 30% (*v*/*v*) aqueous solution of trichloroacetic acid was added, and the resulting solution was vortexed for about 5 s and filtered with Whatman No. 1 filter paper. Five millilitres of the filtered solution were mixed with an equal volume of 0.02 M thiobarbituric acid aqueous solution, placed in a hot water bath at 90 °C for 20 min, and then refrigerated. After centrifugation at 4500× *g* for 5 min, the absorbance of the supernatant at 530 nm was read spectrophotometrically. Solutions of 1,1,3,3-tetramethoxypropane at concentrations between 0.016 and 0.165 µg/mL were read to construct the calibration curve (R^2^ = 0.99).

### 2.7. Sensory Evaluation

EY-1 and EY-2 yoghurts were sensory evaluated by a panel of 19 judges composed of 10 males and 9 females aged between 25 and 62 years old. The evaluation of odours, tastes, and appearance was performed according to a list of eleven attributes: odour intensity and unpleasant odours (odour perception); sweet, acid, bitter, persistence, and off flavour (taste sensation); and colour, homogeneity, spontaneous syneresis, and viscosity (appearance and texture). The judges were also asked to give an overall evaluation in terms of satisfaction with the final product, considering the scores of all attributes [20,43,44]. To this purpose, the members of the descriptive panel used a sensory 9-rating scale as reported by Gaglio et al. [27], where 0 corresponds to the lowest score of the character and 9 to the highest value. Sensory assessment was performed in individual temperature-controlled cabs (20 °C). A commercial white cow’s yoghurt (CCY) (Muller, Fischach, Germania) purchased at a retail market was used as a control. Each yoghurt (35 mL) was served at 7 °C using plastic cups coded with an alphanumeric random code. Water was used for rinsing between samples that were randomly presented as described by Akalın et al. [45].

### 2.8. Statistical Analyses

The data were analysed using XLStat software version 2020.3.1 for Excel software (Addinsoft, New York, NY, USA). A statistical analysis of bacterial counts was performed after logarithmic conversion of the data. Results are given as means ± standard deviation (SD). Differences between the means were determined by Tukey’s multiple range post hoc test. *p* values of < 0.05 were deemed to be significant.

The effect of starter culture on the physicochemical traits of yoghurt was evaluated statistically using the generalised linear model (GLM) procedure in SAS 9.2 software (SAS Institute Inc., Campus Drive, Cary, NC, USA).

## 3. Results and Discussion

### 3.1. Microbiological Monitoring

**Figure 2:**
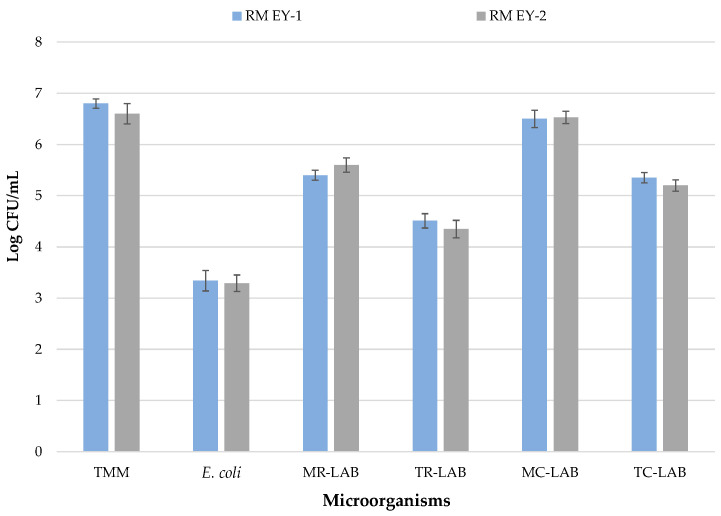
Microbiological loads of milk samples before pasteurization. Results indicate mean values and SD of four determinations (carried out in duplicate for two independent productions). Abbreviations: EY-1, production performed with freeze-dried commercial starter culture (starter formulation YODOX91); EY-2, production performed with a multi-strain milk starter culture consisting of *S. thermophilus* and *L. delbrueckii*; RM, raw milk; TMM, total mesophilic microorganisms; *E.*, *Escherichia*; LAB, lactic acid bacteria; MR, mesophilic rod; TR, thermophilic rod; MC, mesophilic cocci; TC, thermophilic cocci.

The results of microbiological analyses of raw milk samples from EY-1 and EY-2 productions are shown in Figure 2.

In both productions, RM samples show no statistically significant differences (*p* > 0.05) for the microbial groups investigated. These samples show levels of TMM at about 10^7^ log CFU/mL in EY-1 and EY-2. Similar levels were registered by Gaglio and co-workers [27], who analysed raw cow’s milk before transformation into yoghurt, while higher TMM levels have been detected in raw cow’s milk in other studies [46,47]. The high TMM cell densities found in raw milk are not surprising since this food matrix, thanks to its high concentrations and variety of nutrients and its neutral pH, represents the ideal growth medium for a wide variety of microorganisms [48]. Raw milk microbiota is very heterogeneous [49] and can be made up of pathogenic and spoilage microorganisms as well as bacteria of technological importance [50,51]. Regarding the specific search for undesired bacteria, plate counts did not detect *L. monocytogenes* or *Salmonella* spp. To date, the regulation (EC) No 2073/2005, which includes microbiological parameters [29], indicates the complete absence of these microorganisms for the human consumption of milk. Indeed, none of the samples show the presence of CPS.

The absence of staphylococci, potential contaminants in raw milk samples, is evidence of good hygiene practices applied during milking, conveyance, and storage [52], because bacterial cross-contamination of milk can arise as a result of many factors [53]. Nevertheless, plate counts revealed the presence of *E. coli* at levels of 3.34 and 3.29 CFU/mL in EY-1 and EY-2 production, respectively. Similar results were found by Caro et al. [54] on samples of raw ewe’s milk used to make “Castellano” cheese. *E. coli* represents an indicator of faecal contamination [55], and such contamination during the milking process is quite common [56,57,58]. For this reason, a sanitization treatment becomes mandatory if the final product has a short maturation period (<2 months) [59].

LAB were dominant in raw milk samples; levels of mesophilic rod and cocci LABs were almost superimposable to those of TMM (log 5.4 and 6.5 CFU/mL, respectively, in EY-1; log 5.6 and 6.53 CFU/mL, respectively, in EY-2). Similar results were previously registered by Samelis et al. [60] for raw milk used for Greek hard cheese production.

Thermophilic LAB were detected at levels lower than mesophilic LAB since log 10^5^ CFU/mL were recorded for thermophilic cocci. Thermophilic rods were even lower (log 10^4^ CFU/mL) in both productions. Garofalo et al. [61] registered a similar trend for raw ewe’s bulk milk collected in a different area (Palermo province) of Sicily. The microbial ecology of raw milk consists of a complex interaction among indigenous LAB, which play different roles during cheese-making [62], and they are generally high in number [63].

**Figure 3:**
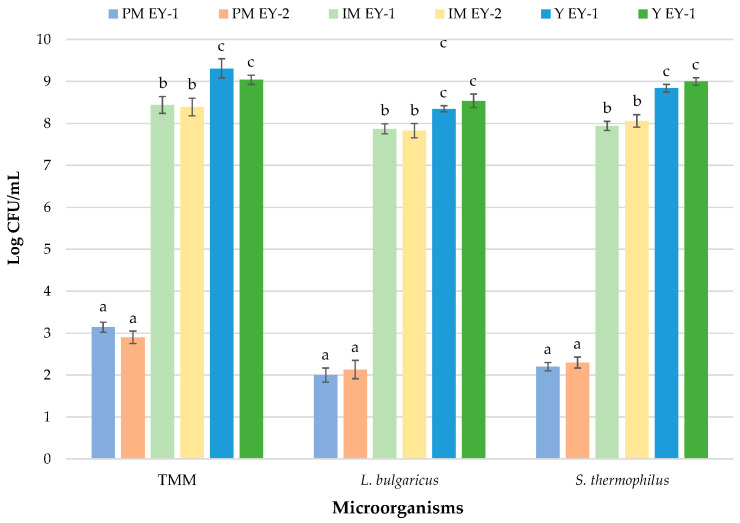
Growth of starter cultures during yoghurt production. Units are log CFU/mL for liquid samples and log CFU/g for solid samples. Results indicate mean values ±SD of four plate counts (carried out in duplicate for two independent productions). a, b, c = *p* < 0.05. Abbreviations: TMM, total mesophilic microorganisms; *S.*, *Streptococcus*; *L.*, *Lactobacillus;* PM, pasteurised milk; IM, inoculated milk; Y, yoghurt; EY-1, production of ewe’s yoghurt performed with freeze-dried commercial starter culture (starter formulation YODOX91); EY-2, production of ewe’s yoghurt performed with a multi-strain milk starter culture consisting of *S. thermophilus* and *L. delbrueckii*.

Inoculated starter cultures drove the fermentation process during yoghurt manufacture. Therefore, microbiological analysis included the detection of thermophilic LAB cocci and rods representing the starter cultures inoculated. Figure 3 shows the results of the microbial growth from pasteurised milk to final yoghurts.

After pasteurization, the levels of TMM in milk were recorded at approximately three log cycles in comparison to RM samples. Pasteurization treatment is necessary to guarantee the hygiene and security of the final products [64]. Figure 3 clearly highlights a decrease of approximately three log cycles for TMM in PM samples. The main aim of thermal treatment (72 °C for 15 s) is to sanitise raw milk from any potential trace contaminants it may have. A sudden and consistent drop was also registered for the levels of LAB and *E. coli*. In particular, *E. coli* was undetectable after the application of the thermal treatment. Although pasteurization causes a drastic reduction in microbial levels, this treatment does not determine the complete elimination of the indigenous microbiota [65]. This treatment leads to a reduction of at least 90% of the bacterial population, and usually only thermoduric bacteria survive [66].

Immediately after starter addition, the levels of *S. thermophilus* and *L. delbrueckii* in the yoghurt were approximately log 10^8^ CFU/g in both productions. It is widely known that starter microorganisms should be added at levels of 10^7^ CFU/g of viable bacteria since they have the primary task to produce lactic acid from lactose [67]. *S. thermophilus* was observed at slightly higher levels than *L. delbrueckii* in IM of both productions, with no statistically significant differences (*p* > 0.05). These results are in line with those argued in the study of Güler-Akin (2005) [68]. After fermentation, the final yoghurts show count loads one log cycle higher than inoculums (*S. thermophilus* and *L. delbrueckii* were detected at 8.84 and 8.35 CFU/g, respectively, in EY-1 and 9.00 and 8.54 in EY-2). Similar results can be found in the work of Shazly et al. [69], who produced probiotic yoghurt from ewe’s milk. Birollo et al. [70] stated that the high levels of viable LAB in fermented milks are correlated with consumers’ health benefits.

### 3.2. Physicochemical Traits of Ewe’s Yoghurt

Chemical composition and colour parameters of milk used for the production of yoghurts (Table 1) show some fluctuations, especially for protein, casein, urea, and yellowness, that can be attributed to the different feeding systems of the origin farms of milk [71]. However, milk parameters fully reflect those characterizing sheep milk from Valle del Belìce sheep [72].

Regarding the physicochemical traits of the yoghurts produced with the different types of starters (Table 2), the differences in protein content can be attributed to the different starting milk composition since the protein percentage changes from milk to yoghurt occurred at similar levels (about +20%) for both production lines.

Compared to milk fat, yoghurt fat shows a greater reduction with the use of NMSC than commercial starter, suggesting an effect of more intense bacterial activity in degrading fatty acids as an energy source [27].

None of the colour parameters of yoghurts (Table 2) were significantly affected by the starter culture, but the use of NMSC induced a significant increase in the total colour change. This result can be explained by the larger change from milk to yoghurt observed in the EY-2 yoghurt than in the EY-1 yoghurt for lightness and yellow index, even if these differences between yoghurts did not emerge at a statistically significant level; these results can be imputable to the microbial activity carried out by the bacteria constituting the selected starter culture.

### 3.3. Oxidation and Antioxidant Activity of Ewe’s Yoghurt

The antioxidant capacity is defined as the action of all the antioxidant molecules present in the food matrix [73]. Authors such as Jaster et al. [74] and Guz et al. [75] describe yoghurt as a potential carrier of antioxidant compounds derived from its enrichment with a matrix containing bioactive molecules such as polyphenols. However, milk and its derivatives constitute a complex mixture of enzymatic systems, proteins, minerals, and vitamins, and all these substances together confer antioxidant capacity [76]. The antioxidant characteristics of milk and dairy products were extensively studied. Thus, studies on cow’s milk and cow’s yoghurt have shown that the fermented version of milk products has a higher antioxidant capacity than milk [77]; this result can be linked to the antioxidant properties of peptides produced from milk protein by the enzymatic activity of inoculated microorganisms [78]. In this regard, there is no study on non-fortified yoghurt made from ewe’s milk. The present study demonstrates that the application of selected bacteria can improve the antioxidant properties of ewe’s yoghurt, contributing to enhanced health benefits for consumers [79].

The antioxidant activity and the primary and secondary lipid oxidation values of the yoghurts processed in this study are reported in Table 3.

The antioxidant activity of yoghurts was evaluated by the TEAC assay in order to verify the effects of the use of selected strains. A better antioxidant capacity emerged in the EY-2 yoghurt (15.41 mmol TE/kg DM) produced with the selected starters *S. thermophilus* PON244 and *L. delbrueckii* WT601; presumably these microorganisms were able to perform more intense proteolytic activity and release peptides with antioxidant properties [76,78]. Future studies on sheep yoghurt should evaluate its antioxidant capacity during storage, since some studies have stated that this property decreases over time [80]. Moreover, since the antioxidant properties of this type of dairy product can derive from different events, more antioxidant assays are opportune.

Lipid oxidation was evaluated by POV and TBARS analyses. Foods with a low peroxide value are generally perceived as having high oxidative stability and a long shelf life [81]. However, the number of peroxides is often affected [82] and increases with storage time [83,84]. In this study, the level of primary lipid oxidation in yoghurts produced using different starters was compared. Higher values were found for EY-2 yoghurt (0.43 O_2_/kg fat), presumably due to a greater microbial activity than that of EY-1, which led to an anticipated oxidation of the product. On the contrary, for secondary lipid oxidation, EY-2 yoghurt shows the lowest value (0.074 MDA/kg DM), and this is attributable to its higher antioxidant capacity than EY-1. In the literature, no other study reports the values of primary and secondary lipid oxidation of ewe’s yoghurt; however, the values observed in this study were analogous to those reported for fresh sheep cheese [61].

### 3.4. Ewe’s Yogurt Sensory Evaluation

**Figure 4:**
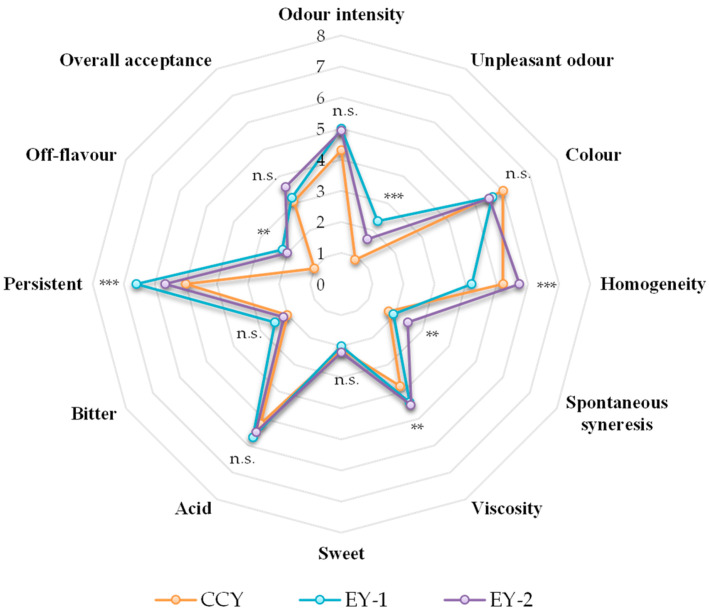
Spider diagram of sensory evaluation of yoghurts. Abbreviations: CCY, commercial cow’s yoghurt; EY-1, production of ewe’s yoghurt performed with freeze-dried commercial starter culture (starter formulation YODOX91); EY-2, production of ewe’s yoghurt performed with a multi-strain milk starter culture consisting of *S. thermophilus* and *L. delbrueckii*; ** *p* < 0.01; *** *p* < 0.001; n.s., not significant (*p* > 0.05).

The panellists scored the three yoghurt samples as objects of evaluation (EY-1, EY-2, and CCY), and the results for all attributes are given in the form of a spider plot (Figure 4). It is well known that the sensory attributes of dairy products are strictly associated with the milk composition, starter cultures, heat treatment, and storage conditions [85]. For this reason, the sensory evaluation of a new dairy product is mandatory before its commercialization in the marketplace [86]. The scores of the assessors for odour intensity, colour, sweetness, acidity, and bitterness were not statistically different (*p* > 0.05) among samples. These results are encouraging because they suggest that ovine milk yoghurts meet the basic requirements of consumers, which, according to Routray et al. [17], are colour, odour, and flavour. These attributes represent the most significant sensory factors used for the promotional marketing of a new dairy product [87]. Statistically significant differences (*p* < 0.0001) observed for unpleasant odour, homogeneity, and persistence in the mouth may be attributable to the type of milk used. Indeed, sheep’s milk is different from cow’s milk in terms of gross composition and single constituents. For example, the lipid fraction contains a higher percentage of volatile, short-chain fatty acids, such as rancid flavour notes [88]. The fat content also serves as substrate for important flavour-generating reactions performed by microorganisms, but it also contributes to the body and texture of cheese, which explains the differences (*p* < 0.01) observed between spontaneous syneresis, viscosity, and off-flavour. Bonanno et al. [89] stated in their work that the differences observed are induced by the breed, the nature of the feed consumed, and even the time of milking (i.e., morning vs. evening). In addition, the use of sheep milk is well known to enrich the nutritional properties of the final product because of the high levels of total solids and nutrients [90].

Finally, the panel was also asked to express their preference between the samples, and even if no difference was noticed between all samples, EY-2 received a higher score (3.6). This means that LAB strains consistently impacted the organoleptic characteristics of the final yoghurts.

## 4. Conclusions

The results of this work show that selected starter cultures can be used to create new and safe products from ewe’s milk, such as yoghurt, with good antioxidant properties. The consumers also liked the sheep’s milk yoghurt, which is essential for its market success. This work explored a novel dairy product for Sicily that can benefit the sheep farmers by preserving the native breeds and biodiversity. In a future perspective, sheep yoghurt can help face the increasing phenomenon of abandonment of inland areas affecting mountain provinces of the main island.

## Figures and Tables

**Table 1:** Composition of milk used for yoghurt production.

Milk	First Yoghurt Production	Second Yoghurt Production
pH	6.07	6.31
Lactose, %	4.33	4.16
Fat, %	6.80	6.23
Protein, %	5.43	4.43
Casein, %	4.04	3.12
Urea, mg/dL	57.00	67.40
Colour		
Lightness L*	91.94	86.35
Redness a*	−3.63	−3.44
Yellowness b*	5.64	3.60
Chroma	6.70	4.97
Hue angle	−57.21	−46.30
Whiteness index	89.52	85.47

**Table 2:** Physicochemical traits of ewe’s yoghurt.

	Samples		SEM	*p*-Value
	EY-1	EY-2		
Dry matter (DM), %	20.30	17.09	0.594	0.0620
Ash, % DM	3.77	4.58	0.149	0.0618
Protein, % DM	35.47	33.04	0.147	0.0072
Fat, % DM	35.07	34.62	0.051	0.0252
Protein change, %	20.27	20.73	0.536	0.6017
Fat change, %	−5.05	−10.03	0.139	0.0016
Colour				
Lightness L*	95.13	94.85	0.521	0.7405
Redness a*	−3.89	−3.59	0.154	0.2964
Yellowness b*	10.25	8.49	0.596	0.1725
Total colour change	5.65	9.84	0.397	0.0175
Chroma	10.96	9.22	0.607	0.1792
Hue angle	−69.21	−67.03	0.626	0.1332
Whiteness index	87.99	89.43	0.578	0.2193

Abbreviations: EY-1, production performed with freeze-dried commercial starter culture (starter formulation YODOX91); EY-2, production performed with a multi-strain milk starter culture consisting of *S. thermophilus* and *L. delbrueckii*. SEM, standard error of the mean.

**Table 3:** Oxidation products and antioxidant capacity of ewe’s yoghurt.

Samples	TEAC, mmol/kg DM	POV, mEq O_2_/kg fat	TBARS, mg MDA/kg DM
IM EY-1	0.63	0.16	0.092
IM EY-2	15.41	0.43	0.074
SEM	0.263	0.011	0.001
*p* value	0.0006	0.0036	0.0087

Abbreviations: EY-1, production performed with freeze-dried commercial starter culture (starter formulation YODOX91); EY-2, production performed with a multi-strain milk starter culture consisting of *S. thermophilus* and *L. delbrueckii*; TEAC, Trolox equivalent antioxidant capacity; POV, peroxide value; TBARS, thiobarbituric acid reactive substances; MDA, malonylaldehyde; SEM, standard error of mean.

## Data Availability

The data that support the findings of this study are available from the corresponding author upon reasonable request.
